# How does digitally enabled micro-finance promote income equality for the vulnerable in the expanded BRICS block during the pandemic?

**DOI:** 10.3389/fdata.2024.1417752

**Published:** 2024-12-02

**Authors:** Manoj Kumar M. V., Nasser Almuraqab, Immanuel Azaad Moonesar, Udo Christian Braendle, Ananth Rao

**Affiliations:** ^1^Department of Information Science and Engineering, Nitte Meenakshi Institute of Technology, Bangalore, India; ^2^Dubai Business School, University of Dubai, Dubai, United Arab Emirates; ^3^Mohammed Bin Rashid School of Government, Dubai, United Arab Emirates; ^4^IMC University of Applied Sciences Krems, Krems an der Donau, Lower Austria, Austria

**Keywords:** income equality, human capital, alternative finance, digital micro-finance, digitization, entrepreneurship, governance, SDG 3

## Abstract

**Introduction:**

Tech-enabled alternative micro-finance promotes income equality in growing BRICS and Austria across financial crises and pandemics. Are financial access and digital skills equally economically valuable? Our study uses inputs: Human Capital, Alternative Micro-finance, Digitization, Governance, and Entrepreneurship, GDP, inflation, population growth, pandemics, and economic crises using the global 2000–2022 to explain income equality using SWIID Gini disposable and market income index as outputs.

**Methods:**

The study uses Principal component analysis for reducing data dimensionality and collinearity. The study uses OLS, Dynamic Mixed Model, and random forest tree, a machine learning technique, as models to model digitally enable micro-finance.

**Results:**

RFT model diagnostics consistently were better than OLS and GMM. Reduced income inequalities resulted from public and private infrastructure investments, government policy interventions to fight pandemics, economic crises, and conflicts, as well as from expansion in GDP.

**Discussion:**

The study concludes that digitally enabled micro-finance plays a crucial role in reducing income inequalities, particularly during times of crisis. Key policy implications include the need for government support in digital infrastructure to enhance financial inclusion. By pooling their resources, the BRICS block can empower micro-finance organizations to ameliorate disruptions from COVID-19 and economic crises.

## 1 Introduction

Using SDG monitoring, the UN aims to share prosperity and less extreme poverty by 2030. Thus, every nation's greatest asset—human capital—is nurtured and empowered. The poor always have income gaps because they need high-quality human capital to work and study. Youth employment reduces poverty and boosts the economy. Pandemics affect digitalization, financial inclusion, and economic equality; this article examines their relationship.

Previous research has examined income inequality (Consoli et al., 2023; Furceri and Ostry, 2019; Iacono and Ranaldi, 2022; Tassaeva, 2021), but digital technology adoption, alternative finance, and income equality during uncertain pandemics in expanded BRICS is lacking. Alternative finance, artificial intelligence, and digital wallets prioritize these links to minimize income inequality through social welfare, notably during pandemics in the expanded BRICS economies.

Despite significant technical growth, the BRICS countries face income inequality. As most of the world's population lives in these countries, technology influences economic inequities, governance, digitization, human capital, and technical advances, which must be addressed. With the focus on inclusive economic growth, financial incorporation may assist these nations in fostering more equitable development. The BRICS alliance expanded to eleven states with US$30.76 trillion in GDP, 30% of the world economy, and 40% of the global population in October 2023. BRICS members emphasized cooperation to advance the SDGs and reduce poverty, food insecurity, and global recovery. Zeroing poverty, especially extreme poverty, is the biggest global issue and a prerequisite for sustainable development. BRICS growth boosts competitiveness through e-skills and finance. Recently, a study suggested that the enlarged BRICS block that prioritized ICT in Smart Specialization is more varied and inventive (BRICS Summit, 2023).

The COVID pandemic in BRICS, with lockdowns and movement restrictions, hindered economic activity and earnings because most micro-finance recipients labor in informal or semi-formal industries. Liquidity shortages increased micro-finance defaults. In such scenarios, micro-finance providers leveraged digital platforms for loan distribution and collection and reevaluated lending criteria and risk assessment methods.

For human capital mobilization, BRICS uses alternative funding for R&D, education, infrastructure, and skill development. Without skill upgrading, digital technology and finance may widen economic divides (Iammarino et al., 2020). Digitalisation and financial inclusion can help economies catch up and lessen inequity gaps, but pandemics may increase income disparity. Income inequality determinants must be identified to overcome future growth constraints (Iammarino et al., 2019). Digital technologies are skill-biased. Therefore, e-skill development may affect labor demand and wages differently for persons with various education, occupations, and employment (e.g., routine vs. non-routine jobs). E-skills allow copying and altering advanced external and financial data. Innovation and creativity will boost revenues for established organizations in some industries and give startups and SMEs new commercial and technology prospects in others. Talent bias and innovation affect workforce and corporate earnings, affecting income distribution.

Antwi et al. (2024) examine how competition and financial inclusion affect financial stability in 60 developing countries from 2002 to 2019. Using the system GMM estimator, their work highlights financial development as a key driver of financial inclusion and financial stability in developing economies. Their research suggests ways to increase financial inclusion in developing economies to improve financial stability.

Bibi et al. (2024) explore how governance affects social inclusion (SI), ICT, and financial inclusion (FI) in 46 countries from 2010 to 2020. Panel-corrected standard errors, fully modified OLS, and dynamic OLS methods were used. SI negatively affects FI. FI benefits from ICT infrastructure. Governance with good ICT infrastructure and inclusive communities boosts FI.

SWIID (Solt, 2020) Gini index from 2000 to 2022 is used as a measure of income inequality. Human capital, financial inclusion, digitization, governance, and entrepreneurship are independent factors to explain income equality. GDP, inflation, population growth, pandemics, and economic crises operate as controls. The expanded 11 BRICS group (Dummy =1) is contrasted to Austria, the EU country with the lowest economic inequality, highest financial access, and highest digitization. We use OLS, GMM and random forest tree (RFT) methods to model the relationships.

RFT ranks BRICS and Austria factors in decreasing significance. Governance indicator, Human Capital indicator, digitization, and alternative micro-finance (AAMF) reduced income disparities, according to RFT model results. Inequality reduction also involves entrepreneurship. Income equality improves with gross fixed capital production (infrastructure investments), GDP, inflation, pandemics, and economic crises. SDG-2030 targets and early research imply that government initiatives to enhance community growth and wellbeing reduce income disparity, consistent with the findings of Zhang and Ben Naceur (2019).

### 1.1 Uniqueness

Despite much research on income gaps and technological innovation, few focus on emerging markets. Research is scant, but financial accessibility may help expand the BRICS block to address the income inequality-technical growth gap. In the BRICS block, little is known about how digitization and financial inclusion affect economic inequality. Our research will also further entrepreneurship, governance, human capital, technology, income equality, and financial inclusion research. Policymakers can use our research findings to accomplish SDGs 3-Good Health and WellBeing, 4-Quality Education, 8-Decent Work and Economic Growth, 9-Innovation and Infrastructure, 10-Reduced Inequalities, and 17-Partnerships in BRICS economies by 2030. Despite existing research on income inequality, there is a significant gap in studies exploring the impact of digital finance on income equality within the expanded BRICS block, especially during crises. Our manuscript has these unique qualities.

### 1.2 Innovation

The study uses AI-based Random Forest Tree (RFT) modeling. It shows its superiority over Dynamic Panel GMM regression and OLS methods used in previous studies. Previous research focused on macroeconomic issues or technical developments, but this study investigates both income disparities.

### 1.3 Research objectives

The research objectives are: to evaluate knowledge and skills in promoting income equality; to analyze the role of alternative micro-finance in fostering economic equality; to assess the extent of digitization in advancing income equality; to evaluate the effectiveness of digital tools in boosting entrepreneurship and earnings equality; and to analyze government policy interventions in promoting income equality and inclusion among SMEs.

## 2 Conceptual framework and literature review

### 2.1 Theoretical framework

#### 2.1.1 Theory of micro-finance

In the research article “Micro-finance: A bibliometric exploration of the knowledge landscape” (Pattnaik et al., 2024) authors explained the key theories such as Economic, Financial Intermediation, Institutional, Information Asymmetry, and Social Capital Theories to model micro-finance. According to these theories, micro-financial institutions (MFIs), sometimes known as “banks for the poor,” provide fundamental financial goods and services at affordable prices to low-income people excluded from the traditional financial system. MFIs provide credit to poor and near-poor borrowers excluded from the traditional banking system, allowing them to become financially included. Accordingly, MFIs can help reduce poverty and boost economic opportunities worldwide. Leveraging innovative contractual structures and organizational forms to lower the risk and expense of small, uncollateralized loans is a priority for the micro-finance movement. Recent studies suggest that digital financial services have the potential to bridge income gaps, yet their impact on the expanded BRICS block remains underexplored.

“The Microfinance Schism” (Morduch, 2000) critiques the “win-win” proposition that microfinance can simultaneously achieve financial sustainability and reduce poverty. He argues that this assumption lacks strong empirical support, especially when it comes to serving the most vulnerable populations. Morduch's insights are crucial when considering how digital microfinance can expand outreach. While digital tools may reduce costs and increase access, there is a risk that the focus on financial sustainability could overshadow social goals, such as promoting income equality for vulnerable groups during crises like the pandemic.

Alternative strategies of for-profit, not-for-profit and state-owned Nepalese microfinance institutions for poverty alleviation and women empowerment (Dhungana et al., 2023) investigates how different types of microfinance institutions (MFIs) in Nepal adopt varied strategies to alleviate poverty and empower women. The study finds that for-profit, not-for-profit, and state-owned MFIs employ distinct approaches, with private MFIs surprisingly reaching poorer populations more effectively than expected. In the context of digital microfinance promoting income equality during crises, such as the pandemic, this research provides valuable insights into how various microfinance strategies target different socioeconomic groups. The lessons from the Nepalese context can inform how digital microfinance, when applied in the BRICS block, can balance financial inclusion with social impacts, particularly for vulnerable populations.

In the research work “How the method for delivering loans impacts on the economic efficiency of micro-finance institutions” (Fernández Sánchez et al., 2024) indicate that village banking and solidarity groups improve MFIs' cost efficiency compared to traditional individual loans. In addition, MFIs with a higher number of rural borrowers are more cost-efficient than those with more urban borrowers since Community financing has an edge over traditional lending in collecting borrower information. MFIs should use cost-efficient lending methods to attain financial self-sufficiency and long-term sustainability.

#### 2.1.2 Collaborative intervention theory of digital financial services

According to Pattnaik et al. (2024), DFS theory states that various stakeholders can achieve income equality. Income equality requires multi-stakeholder collaboration (Yunus et al., 2010). DFS's actions expand digital financial systems by delivering financial services to more customers. Financial institutions, commercial banks, MFIs, government programs, village savings and loan associations (VSLAs), mobile network operators, self-help groups, and technology corporations provide digital financial services. Technology is popular in East Africa and South Asia, while other regions are still catching up (Morduch, 2000). Technology, finance, and telecom industries collaborate to promote economic equality using digital financial solutions. In socially inclusive societies, people use formal financial services more, which improves income equality (Al-Azzam and Charfeddine, 2022).

Mobile platforms improve operational efficiency and bring financial services to remote places, creating a more inclusive digital economy (Zhang et al., 2022). One important topic of research is the adoption of developing technology in micro-finance. How these technologies can connect financial service providers to the financially excluded population offers new growth and service delivery prospects (Vassallo et al., 2019). The problems and effects of integrating digital technologies, including cashless digital payment services, peer lending, crowdfunding, and others, are crucial for researchers, industry stakeholders, and policymakers (Daowd et al., 2021). Technology and data can help MFIs become customer-centric, reduce operational risks, cut costs, and boost efficiency (Wondirad, 2022).

#### 2.1.3 Theory of income inequality

Inequality undermines economic growth and stability. Based on Schumpeterian Growth Theory, Aghion (2002) argues that innovation and technological advancement may initially increase between groups, but they can eventually promote upward mobility and reduce inequality by investing in education and skills. Ahn et al. (2018) showed that income disparity affects consumption spending, and low-income households react slowly to economic shifts, according to their models. These studies show that income disparity affects economic growth and redistribution. Ahmed and Shadmani (2024) show that government transfer lowers female income inequality. While the female income ratio rises, government transfer outlays fall throughout the anticipated horizon.

### 2.2 Theoretical research gaps

Although previous literature provides significant insights, the dynamic linkages between income inequality, unemployment rates, and government transfer policies are little understood. Risk protection, simple access to health services, sufficient, affordable education and skills, self-learning capacity, and effective governance through policy action need to be researched to reduce Income disparity.

**Figure 1:**
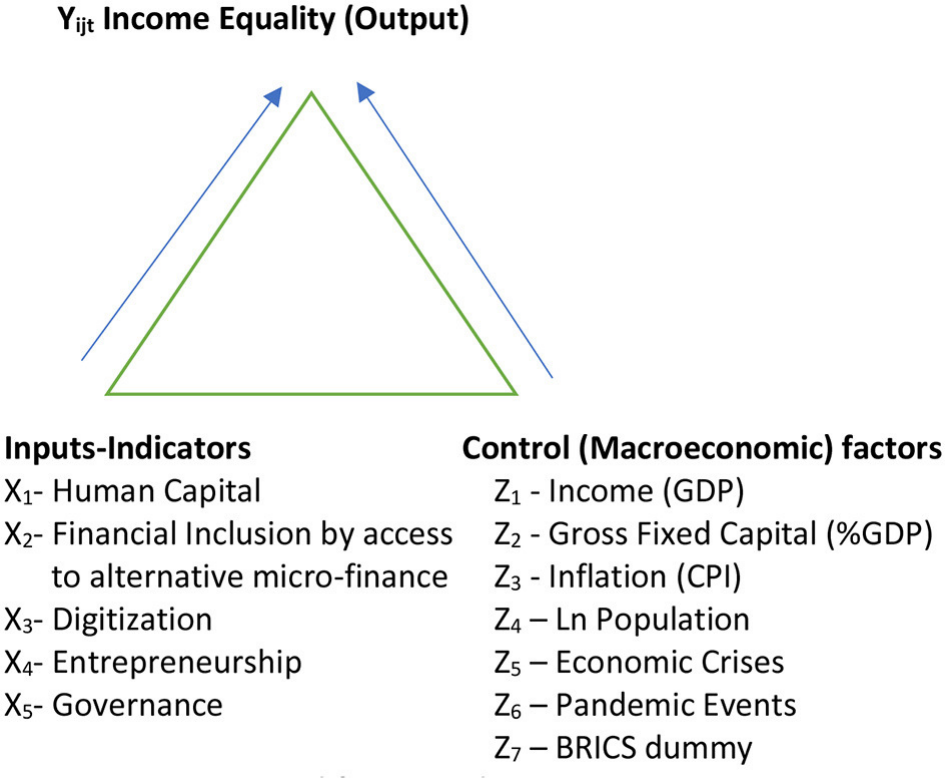
Conceptual framework.

With Austria (EU) serving as the benchmark, our goal is to model income disparity (the output Y_it_) in the enlarged BRICS block and how different explanatory factors (the inputs X_it_ and the control factors Z_it_) impact income equality. Figure 1 illustrates the concept to answer the research objectives. In Figure 1, income equality is conceptually driven by human capital, financial inclusion, digitization, entrepreneurship, governance, and macro-economic factors such as GDP, Inflation, Population, and pandemic events.

Equation 1 shows the empirical formulation of our conceptual framework.


(1) $$\begin{array}{r} {\text{Y}}_{\text{ijt}}=\;\alpha_{\text{ij}}+\;\beta_{\text{ij}}{\text{Y}}_{\text{i}-\text{k},\text{jt}}+\;\pi_{\text{i}-\text{k},\text{j}}{\text{X}}_{\text{i}-\text{k},\text{jt}}+\;\mu_{\text{i}-\text{k},\text{jt}}{\text{Z}}_{\text{i}-\text{k},\text{jt}}\\ +\text{BRICS dummy}+\varepsilon_{\text{i}-\text{k},\text{jt}} \end{array}$$

Where:

- α_ij_ is the constant term, providing the baseline level of the dependent variable when all independent variables are zero.
- β_ij_ measures the effect of the k-lagged dependent variable, Y_i − k, jt_, capturing the persistence or carry-over effect from the previous period.
- π_i − k, j_ and μ_i − 1, jt_ are coefficients for the k-period lagged independent variables X_i − k, jt_ and control variables Z_i − k, jt_, respectively. These variables include critical factors such as Human Capital (X_1_), Financial Inclusion—Access to Alternative Finance (X_2_), Digitization (X_3_), Entrepreneurship (X_4_), and Governance (X_5_), along with control variables that account for other influencing factors.
- BRICS dummy j (Austria = 0 and BRICS = 1) is included to control for specific effects and the collective influence of being a part of the BRICS consortium.
- ε_i − k, jt_ is the error term, accounting for unobserved factors affecting the dependent variable.

We use the Dynamic Panel GMM Model and Machine Learning (ML) methods for estimating the model parameters with *k*-period lagged output, input, and control factors to allow for gradual adjustment over *k* year(s). These are discussed in Section 3.

### 2.3 Interdependence of knowledge-skill competencies and income equality

Human capital affects income equality through knowledge and skills. The human capital index (HCI) measures people's options to live their best lives. In their inaugural Human Development Report (Huang et al., 2023), UNDP identified three important indicators for a decent and sustainable existence: a sensible lifestyle, an education, and a long and healthy life. Due to poorer literacy and unemployment, income inequality diminishes human capital (Le Caous and Huarng, 2020; Alvarado et al., 2021). Weak institutions, unequal financing conditions, population expansion, a loss in human capital investment, a premium on technical know-how, and financial globalization contribute to income disparity in emerging nations, according to Menyelim et al. (2021). When loan markets are weak, starting wealth hinders knowledge and skills investment and increases income inequality (Kunawotor et al., 2020).

### 2.4 Interdependence of financial inclusion (by access to alternative micro-finance) and income equality

According to Konte and Tetteh (2023) and Shaikh et al. (2023), mobile phone availability and formal financial service use are positively connected in developing nations. Lashitew et al. (2019) and Hasan et al. (2022) studied how technology affects financial inclusion. Digital services like mobile money greatly improved traditional financial services' availability and accessibility. The research also noted how technology may lower financial service prices and expand access for low-income consumers. The World Bank (2022) shows how technology is making emerging countries more financially inclusive. Mobile money services make reaching underserved communities easier. This allows them to acquire financial services and conduct transactions without a bank branch (Kass-Hanna et al., 2022; Kouladoum et al., 2022; Demirgüç-Kunt et al., 2022). Technology diffusion increases financial inclusion and reduces financial inequalities (Jalal et al., 2023). Uneven access to finance in rural and poor communities was caused by high financial service costs (Bekele, 2023). These costs include administrative overhead and branch location investments. Due to rising demand for mobile and digital financial services, these expenses have decreased, making it easier for MFIs to operate in remote areas and help marginalized groups (Coffie and Hongjiang, 2023). Financial institutions can tailor financial solutions for underserved or rural communities using customer spending trends. With a better understanding of local financial behaviors and preferences, financial service providers can build services that better serve this market, increasing industry inclusion (Demir et al., 2022; Chen et al., 2021). According to Bansah and Mohsin (2023), credit and savings alternatives help distribute income evenly. Financial inclusion helps people and households develop wealth and minimize income inequality. Financial inclusion improves socioeconomic indices in emerging economies, according to Kim (2022) and Koomson and Danquah (2021). Financial inclusion improves health and education, which helps distribute money more fairly, according to their research. According to Yin and Choi's (2023) analysis, low-quality goods and services can hurt revenue equality in developing countries' monetary integration. Dependence on uncontrollable borrowing might increase poverty.

### 2.5 Interdependence of digital skills and income equality

Digitalization is spreading to finance, healthcare, manufacturing, education, defense, retail, and recreation (Consoli et al., 2023). Digitalisation increases information availability, productivity, innovation, and economic growth (Cardona et al., 2013; Brynjolfsson and McAfee, 2014). ICT network effects reduce entrance barriers and rent-seeking, say Antonelli and Gehringer (2017). More countries that invested in ICT infrastructure gained from component to content and application. ICT expansion drastically reduced skill and education return gaps, lowering bottom-end labor market employment prospects in real earnings (OECD, 2015). To master the vast number of digital technologies and applications, the correct understanding of R&D, management, production, consulting, marketing, sales, and ICT system servicing e-skills is needed for growth. According to Poliquin (2020) and Tewathia et al. (2020), digital skill development is cumulative, and some workers may have more opportunities to improve their income.

### 2.6 Interdependence of entrepreneurship and income equality

As they facilitate innovation, digital skills are equally vital for the effective completion of current activities. To produce new goods and services, these abilities specifically make it easier to acquire, copy, and combine information from other sources. Two different outcomes arise from the creative destruction linked to innovation and digital capabilities. As per Aghion et al. (2019), innovations have the potential to worsen the distributional consequences for workers by strengthening the position of major incumbents and MSMEs and increasing mark-ups. However, by reducing startup costs, closing the opportunity gap between large incumbents and MSMEs, and eventually reducing income disparities among workers, digitization applications can promote entrepreneurship and small enterprises' entry into new markets (Consoli et al., 2023).

### 2.7 Interdependence of governance and income equality

Tchamyou et al. (2019) examine financial services accessibility, ICT, and inequality in 48 African nations. As digital money increased, wealth disparity decreased.

### 2.8 Relevance of control variables with income equality

#### 2.8.1 GDP per capita

In our study, this variable is in constant 2015 U.S. dollars and is expressed in natural Log (Ln). The higher the GDP per capita, improves the income equality.

#### 2.8.2 Total population

Whether due to immigration or more births than deaths, population growth can influence social infrastructure and natural resources. Therefore, depending on how mobile the population is, there might be both good and negative effects on reducing economic inequality.

The final control variable is pandemic events (PE) and economic crises (EC). What impact did these events have on income equality is the study question. The following pandemic occurrences are included in PE: SARS in 2002–2004, EBOLA in 2004, Dengue in 2006, Swine Flu (H1N1) in 2010, Zika in 2015–16, and COVID in 2019–21. EC is the sub-mortgage financial (economic) crisis in 2007–09 and uncertain events such as the Ukraine conflict (2022–24) and the Israel conflict (2023–24). The values of the EC-PE dummies are PE = 1, EC = 1, None = 0.

## 3 Methodology and data indicators

### 3.1 Principal component analysis

**Figure 2:**
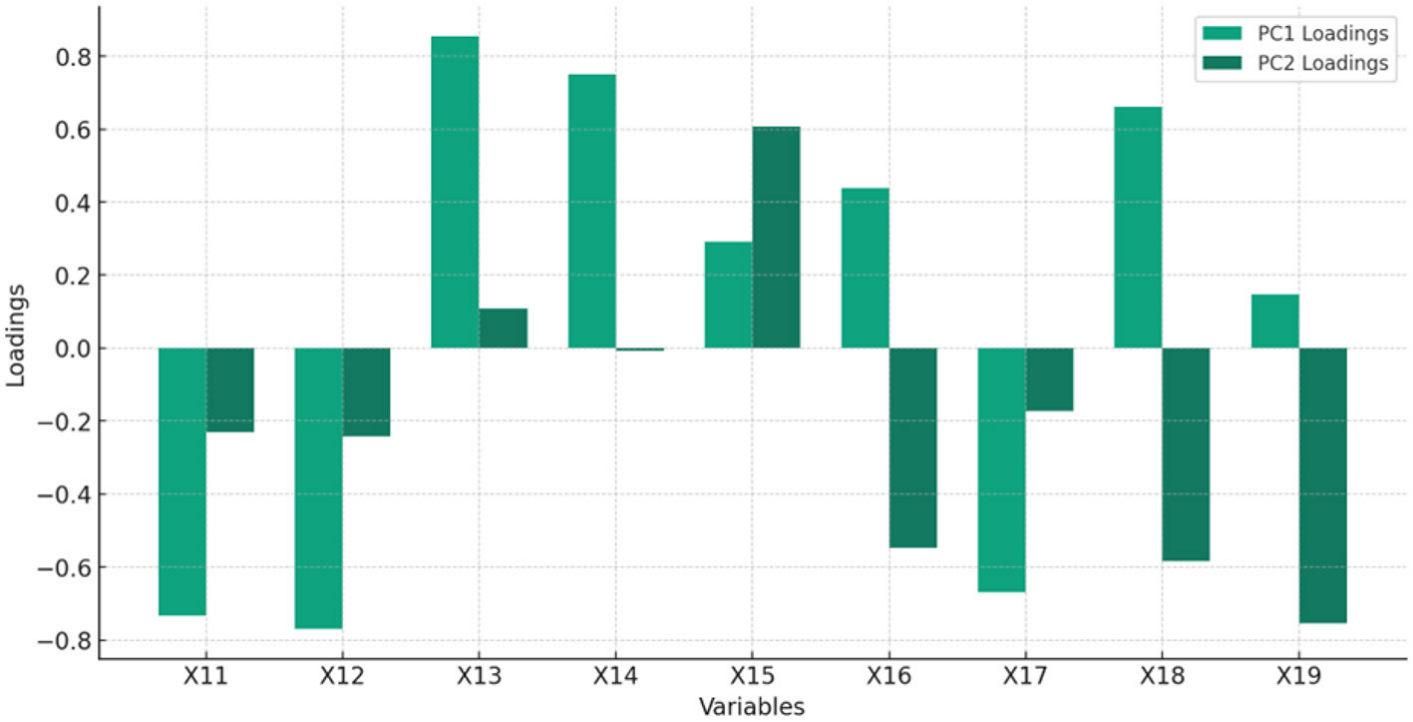
The Percentage of total variance explained by each principal component, highlighting the importance of each component in capturing the dataset's variability related to HUMAN CAPITAL (HC) (these values are detailed in Table 2).

**Figure 3:**
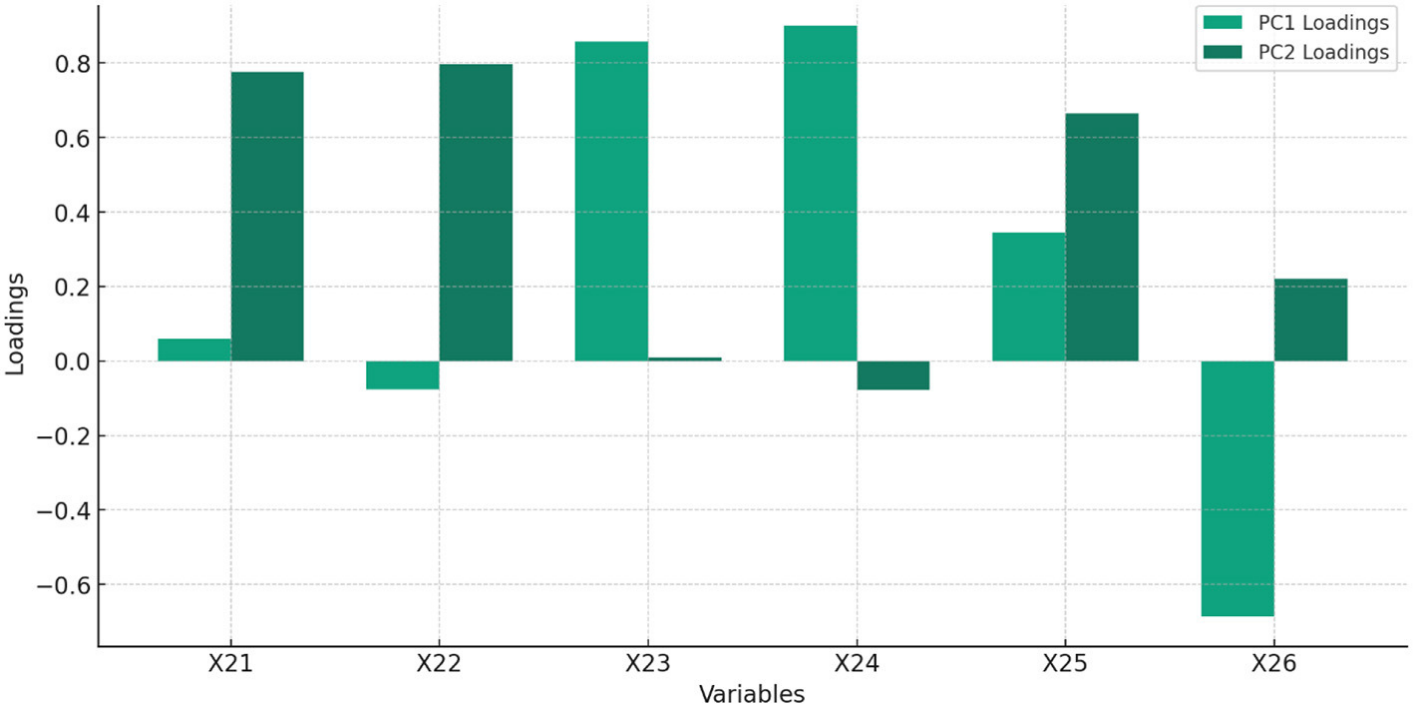
The % of total variance explained by each principal component, highlighting the importance of each component in capturing the dataset's variability related to FI–Access to AAMF.

**Figure 4:**
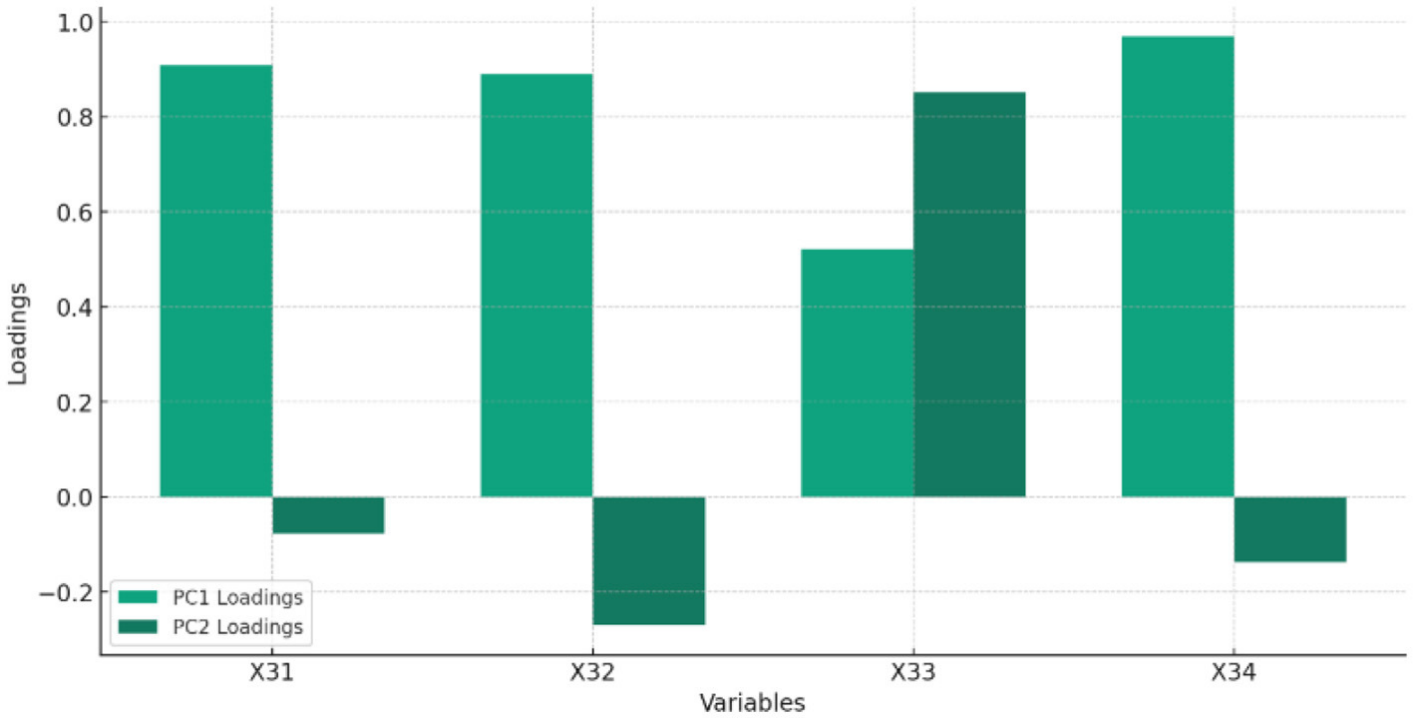
The Percentage of total variance explained by each principal component, highlighting the importance of each component in capturing the dataset's variability related to DIGITIZATION.

**Figure 5:**
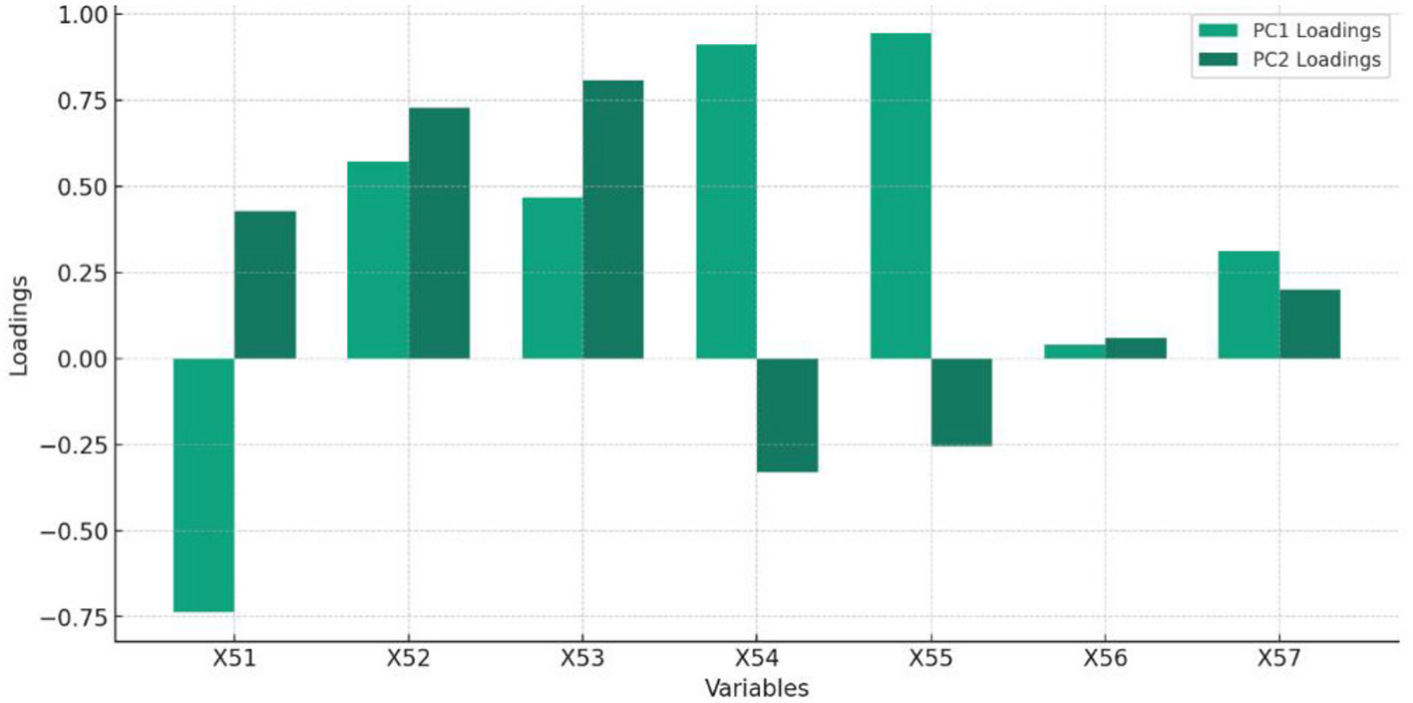
The Percentage of total variance explained by each principal component, highlighting the importance of each component in capturing the dataset's variability related to Entrepreneurship.

**Figure 6:**
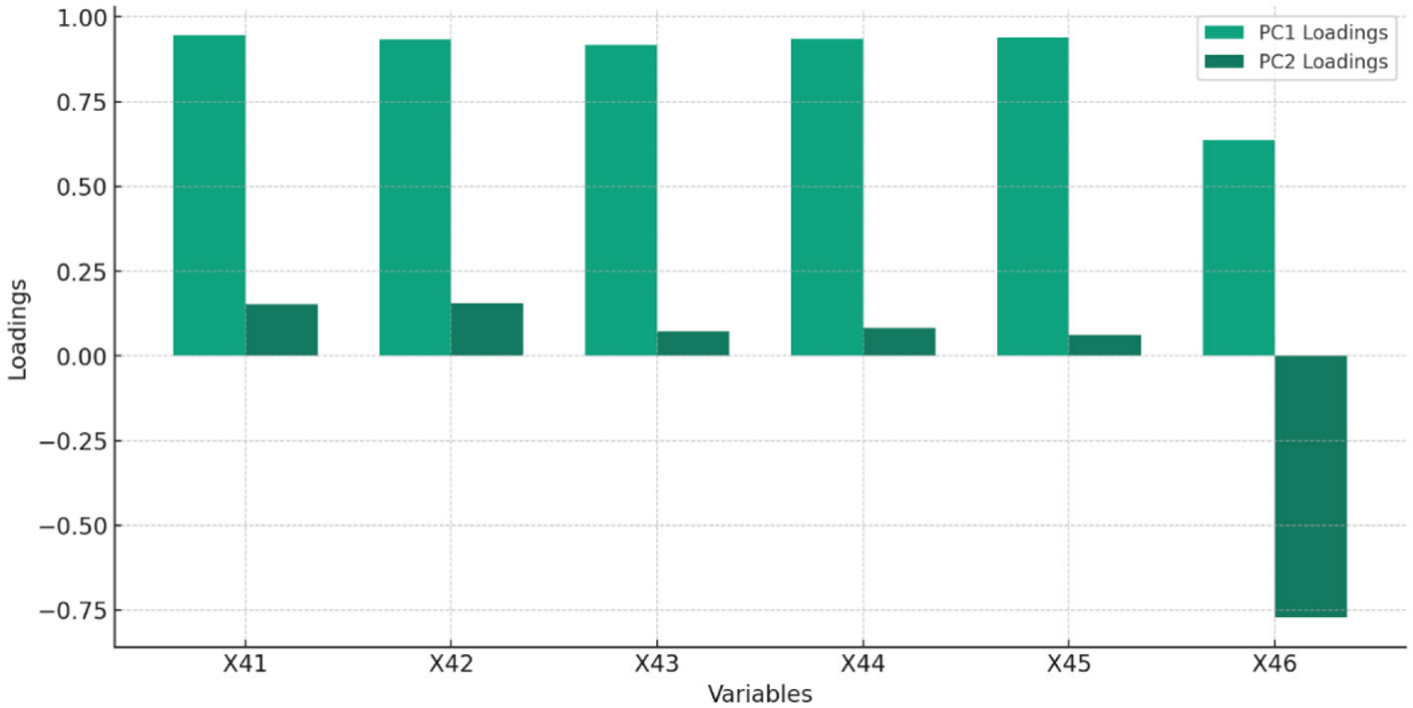
The Percentage of total variance explained by each principal component, highlighting the importance of each component in capturing the dataset's variability related to GOVERNANCE.

**Figure 7:**
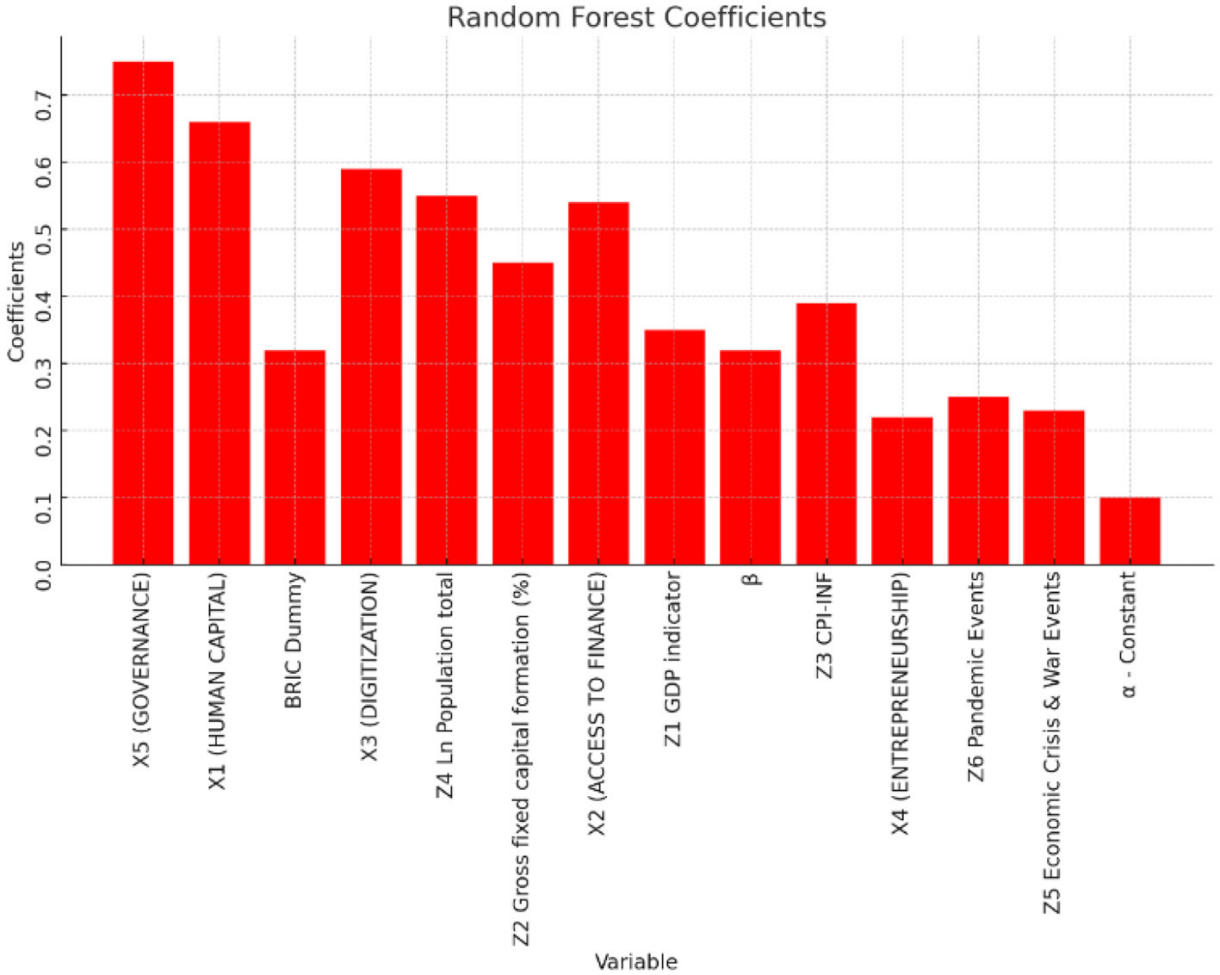
RFT feature importance of the Gini index (Y_1_).

**Figure 8:**
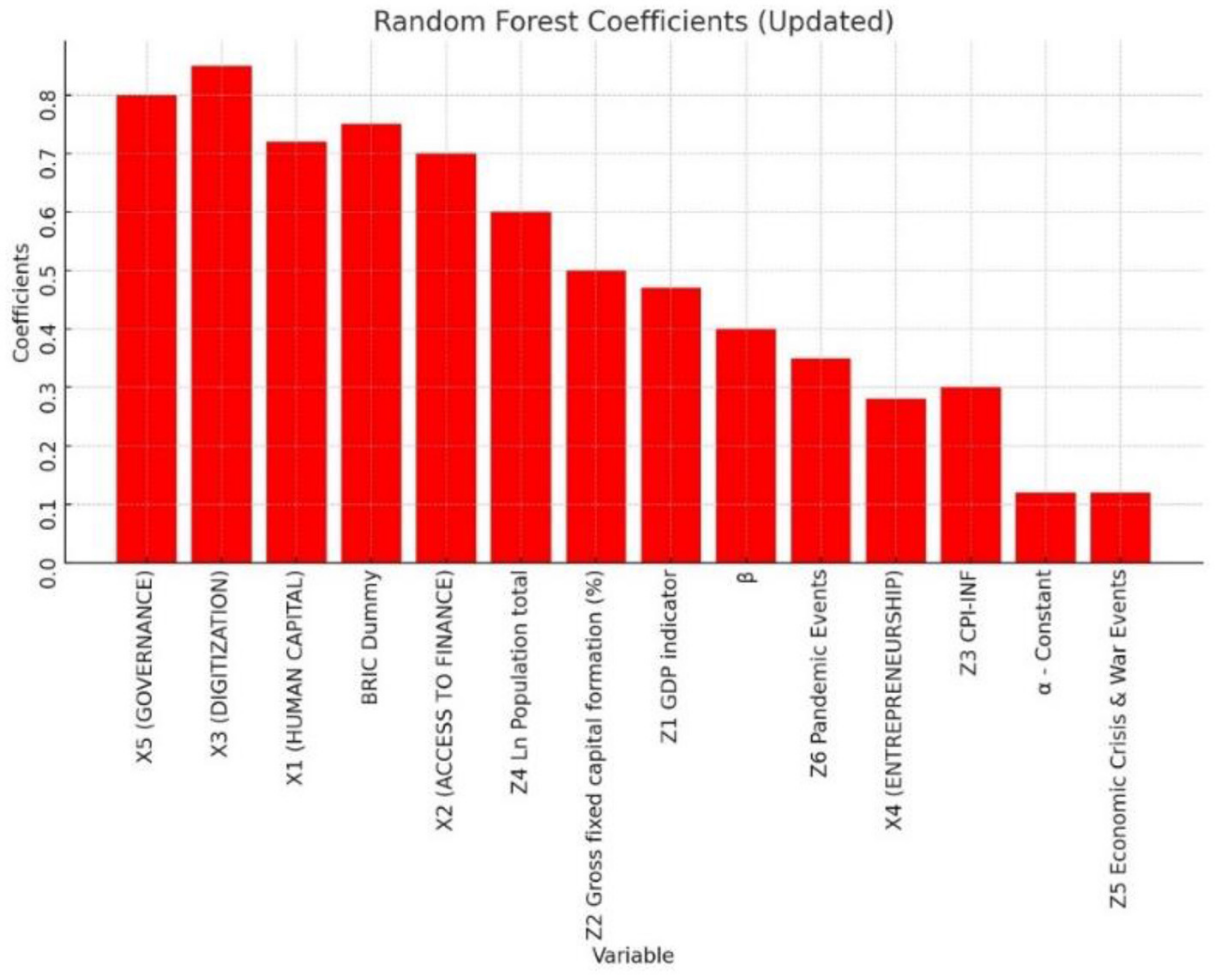
RFT feature importance of the market–based Gini Index (Y_2_).

Principal component analysis (PCA) uses an orthogonal transformation to uncorrelated associated variables. PCA dominates exploratory data analysis and predictive model machine learning (ML). An unsupervised PCA learning algorithm investigates variable associations. A dataset's dimensionality is reduced via PCA without harming the target variables while preserving the most important patterns or relationships by identifying a new set of variables that contain most of the sample's information and help with data regression and classification. The following are features of PCA:

- In decreasing order of significance, principal components (PC) are linear combinations of the original variables in the dataset. PC variance captures equal the dataset's variance.
- **Tables 2**–**7** in Section 4 show that the first PC leads in data variation, whereas the second PC has the most variance orthogonal to it.
- PCA has several applications. PCA plots high-dimensional data in two or three dimensions for data visualization and analysis (see Figures 2–**6** in Section 4). In feature selection, PCA can identify a dataset's most important variables (**Figures 7**, **8**).
- PCA assumes information is contained in feature variance, with bigger variations containing more information.

Given the complexity of economic and social linkages, predicting the impact of digitally enabled micro-finance on income equality requires advanced statistical and ML methods. OLS, GMM, and Random Forest are three popular methods for such an analysis, each with their benefits. Our analysis framework estimates the model in Equation 1.

*OLS* minimizes the sum of squared differences between observed and anticipated values to evaluate the connection between dependent and independent variables. Adjusting for other parameters, OLS can reveal linear relationships between digital financial services availability and use besides various outcomes in digitally enabled micro-finance.

### 3.2 Gaussian mixture model

Gaussian mixture model (GMM) probabilistic model represents subpopulations in a population without knowing which subpopulation a data point belongs to. GMM is an effective way to study the complicated dynamics of digitally enabled micro-finance and its effects on income equality, particularly in the heterogeneous BRICS block and Austria. GMMs capture the heterogeneity of micro-finance data, which comes from several sources and has multiple patterns or distributions. GMMs also reveal latent variables that deeply affect results. Examples include digital literacy, infrastructure, and micro-finance's ability to promote income equality. Because digitally enabled micro-finance has different effects in different nations, GMMs can accommodate different distribution shapes and sizes. GMMs allow the modeling of a mixture of components with distinct means and variances, providing a nuanced and adaptable framework for analyzing the varied effects of micro-finance initiatives and uncovering strategies for reducing income inequality in BRICS+ economies. GMM effectively handles endogeneity difficulties, which occur when independent factors are connected with the error term. The study of digitally enabled micro-finance can benefit from causal inference using GMM's instrumental variables. It enhances understanding of digital financial inclusion's dynamic effects on economic outcomes.

### 3.3 Decision tree model

Decision trees can illuminate numerous key characteristics of digitally enabled micro-finance in BRICS+ and Austria. The models' simplicity and interpretability make them accessible to stakeholders with various technical backgrounds. Digitally enabled micro-finance, where understanding the interaction of causes reducing income equality is vital, benefits from this capability. Secondly, decision trees can handle both qualitative and quantitative data, including micro-finance data like digital platforms, regulatory settings, loan payback rates, and income levels. Thirdly, digitally enabled micro-finance involves identifying characteristics like digital service accessibility, regulatory frameworks, and financial literacy that reduce income inequality. Without linear assumptions, decision trees can model complicated non-linear relationships between variables. Understanding how micro-finance affects income disparities in the actual world requires this competence. Fourthly, decision trees allow for scenario analysis flexibility, allowing for a thorough examination of micro-finance implementation techniques. Adjusting model variables and thresholds lets stakeholders predict policy, technological, and market outcomes. Policymakers and analysts using digitally enabled micro-finance initiatives to reduce bloc-wide income inequality benefit from this versatility.

### 3.4 Random forest tree

**Figure 9:**
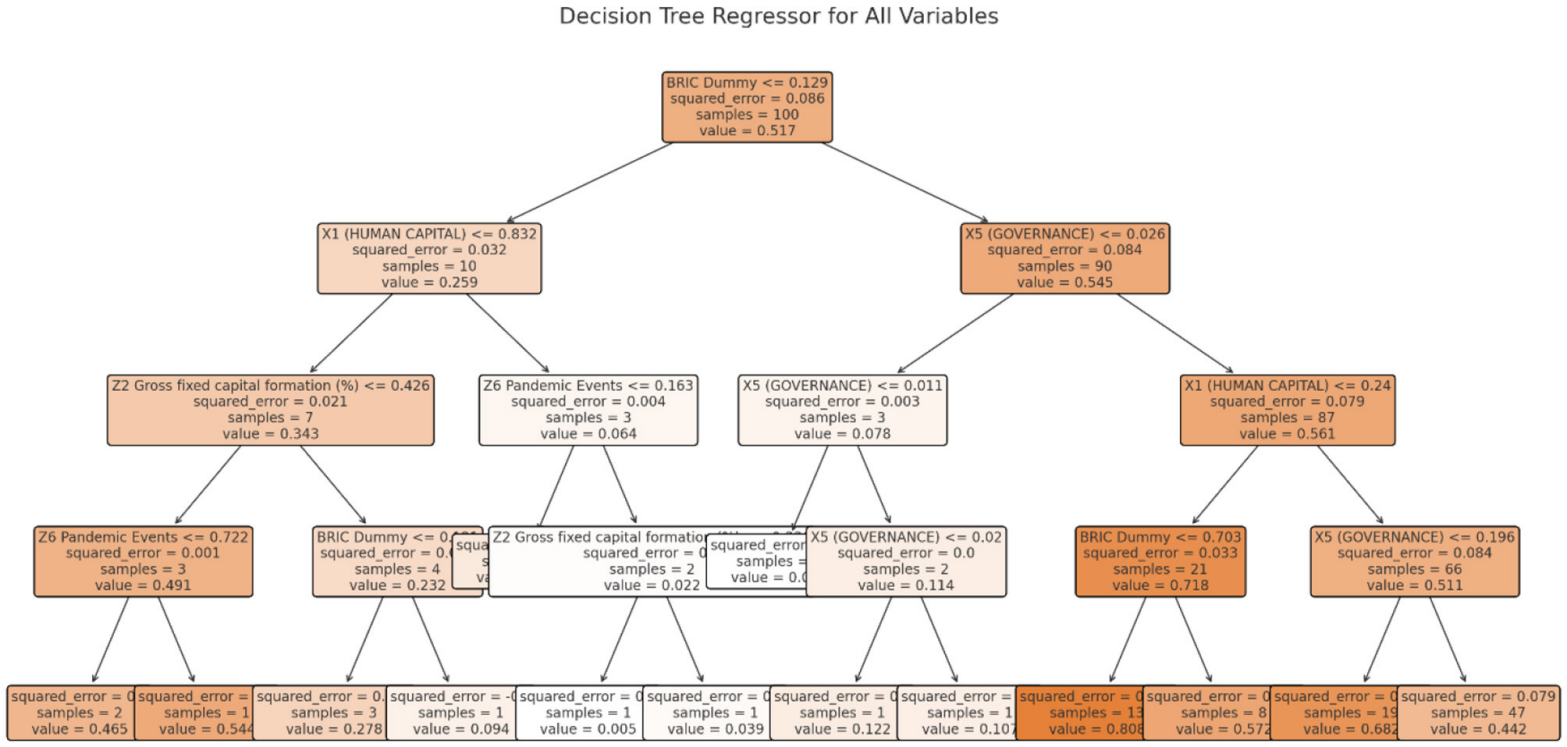
Sample of RFT decision tree for Y_1_.

**Figure 10:**
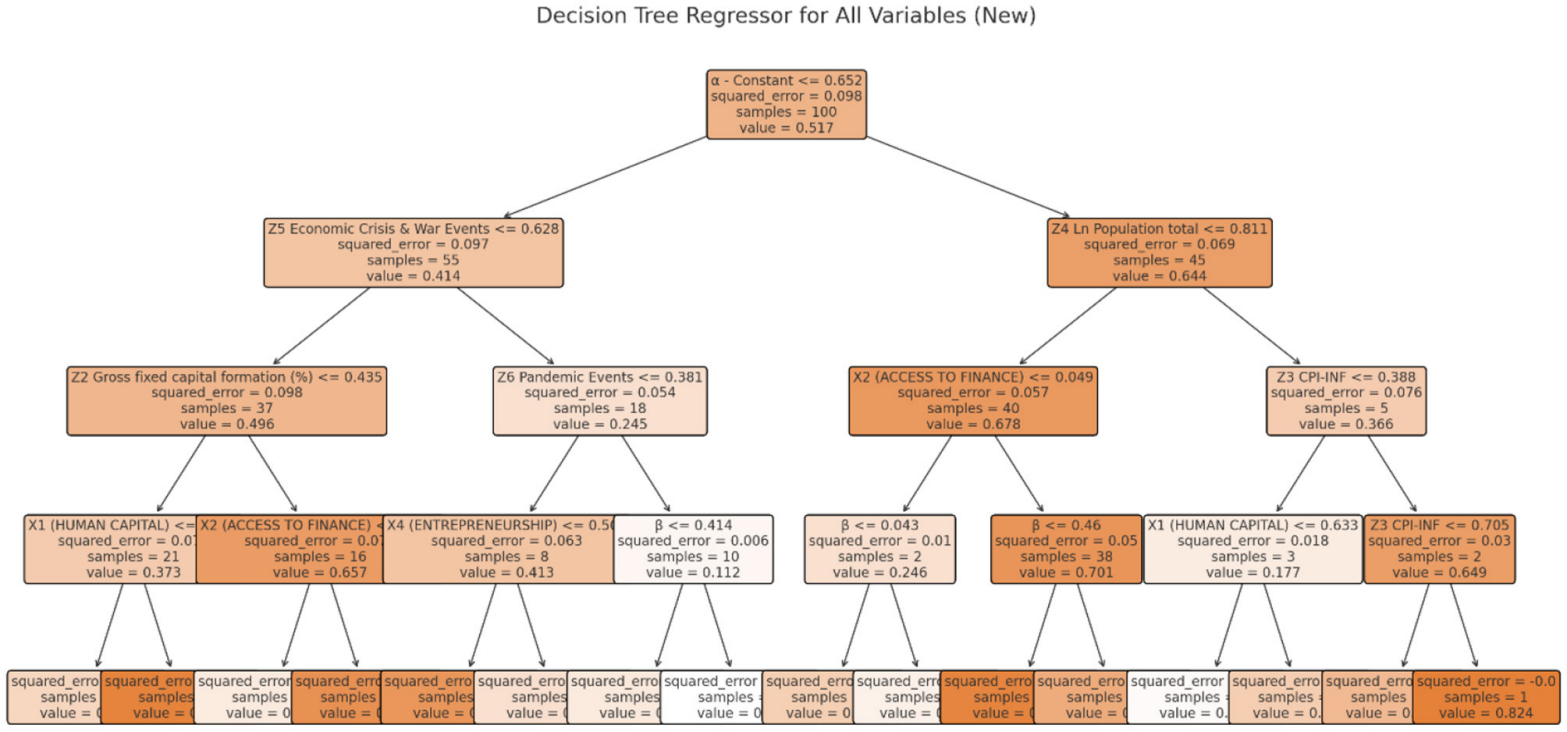
Sample of RFT decision tree for Y_2_.

The Random forest tree (RFT) machine learning method integrates multiple decision trees to improve accuracy and reduce overfitting. It excels at handling non-linear connections and variable interactions. For digitally connected micro-finance, Random Forest can reveal complicated patterns and variable importance beyond typical econometric models. For succinctness, **Figures 9**, **10** show sample RFT decision trees for both dependent variables.

### 3.5 Data indicators

The use of PCA, OLS, GMM, and RFT is grounded in previous research methodologies that effectively analyze complex, multi-dimensional economic relationships. Table 1 shows dependent and independent indicators with sub-variables. The Gini disposable income index, the first dependent variable, shows income concentration or dispersion within the BRICS+ block and Austria. Zero indicates a society with equal income. However, index 1 is a SWIID extreme inequality. Our second dependent variable is the Gini Market Income Index, which includes beneficiary government handouts. For all worldwide economies, SWIID develops both variables. They are alternatives for model robustness testing.

**Table 1:** Summary of Output (Y_ijt_) and Inputs (X_i−k1jt−k_ and Z_i−kjt_).

Gini–Disposable (SWIID Series) income index	Y_1_	**1st Dep. variable**
**Gini–Mkt based (SWIID series) income index**	Y_2_	**2nd Dep. variable**
Advanced education–related unemployment (percentage of the total labor force with advanced education)	HC	**X**_**1**_ **HUMAN CAPITAL**
Female labor force unemployment with advanced education (percentage of women with advanced education)	HC	
R&D Expenditure % GDP	HC	
Researchers in R&D (per million people)	HC	
Patent applications, residents	HC	
Compulsory Education Years	HC	
Fertility rate (births per woman)	HC	
Health Spending as a percentage of GDP	HC	
Spending on education as a percentage of GDP	HC	
Automated teller machines (ATMs) (per 100,000 adults)	FI–AF	**X**_**2**_ **Financial inclusion—access to alternative finance**
Commercial bank branches (per 100,000 adults)	FI–AF	
Borrowers from commercial banks (per 1,000 adults)	FI–UF	
Commercial bank depositors (per 1,000 adults)	FI–AF	
Index of credit information depth (0 being low to 8 being high)	FI–AF	
Legal rights index strength (0=weak to 12=strong).	FI–AF	
Fixed broadband subscriptions (per one hundred people)	DS	**X**_**3**_ **digitization**
Investment in ICT with private participation (current US$)	DS	
Mobile cellular subscriptions (per one hundred people)	DT	
Investment in ICT by public–private partnerships (current US dollars)	DT	
Secure servers for the Internet (per million users)	DT	
People who use the Internet (percentage of the population)	DT	
Ease of doing business score: 0 represents poor performance, 100 represents excellent performance.	Ent	**X**_**4**_ **entrepreneurship**
Women who work for themselves (percentage of employed women) (modeled ILO estimate)	Ent	
Men who work for themselves (percentage of employed men) (modeled ILO estimate)	Ent	
Firms using banks to finance investment (% of firms)	Ent	
Firms using banks to finance working capital (% of firms)	Ent	
Foreign direct investment, net inflows (% of GDP)	Ent	
Portfolio equity, net inflows (current US$)	Ent	
Corruption control (percentile ranking)	Gov	**X**_**5**_ **Governance**
Effectiveness of government: percentile ranking	Gov	
Political stability with the lack of terrorism or violence: percentile rank	Gov	
Quality of regulation: percentile rank	Gov	
Rule of Law: rank in percentage	Gov	
Accountability and voice: percentile rank	Gov	
GDP growth (annual %)	GDP	**Control Z**_**i**_ **variables Z**_**1**_ **GDP indicator**
GDP per capita growth (annual %)	GDP	
GNI growth (annual %)	GNI	
GNI per capita growth (annual %)	GNI	
GDP per person (US dollars constant, 2015)	GDP	
Ln GDP (2015 US$ constant)	GDP	
Gross fixed capital formation (% GDP)	GFCF	**Z**_**2**_ **Ln Infra investments**
Consumer Purchase Index CPI–Inflation	INF	**Z**_**3**_ **Inflation**
Ln Population total	POP	**Z**_**4**_ **Ln Population**
Economic Crisis (EC) (2008–09) economic and financial crises.	EC	**Z**_**5**_ **– EC**
Pandemic events (PE)	PE	**Z**_**6**_ **– PE**
**BRICS (expanded) Dummy: Austria** **=** **0; BRICS** **=** **1**		**Z**_**7**_ **BRICS dummy**
**Year (21 years) used for model estimation; Year 2022 and 2023 used for model prediction**		**Year (2000–23)**

HC, human capital; FI, financial inclusion; AF, access to alternative finance; UF, uses of finance; DS, digital skills (digitization); DT, digital technology (digitalization); Ent, entrepreneurship; Gov, governance; Ln, natural log. Supplementary Appendix 1 contains the details of data sources and early researchers who used them.

### 3.6 Independent variables

#### 3.6.1 X1 human capital indicator

Careers change with industry or local needs, employment requirements, and skills (Vona and Consoli, 2015; Consoli et al., 2023). Digitization tools are the trade's instruments; thus, we expect that directly analyzing employees' talents will give a more accurate indicator of their involvement. The following HC indicator's components demonstrate how HC dimensions affect income equality.

- Compulsory education years: At all ages and income levels, knowledge and skills competencies increase. This helps people earn more and minimizes income inequality.
- The R&D expenditure as % GDP: This includes current and capital R&D expenditure by resident enterprises, research institutes, universities, and government laboratories. Domestic enterprises' R&D abroad is excluded. R&D is “creative work undertaken on a systemic basis to increase the stock of knowledge, including knowledge of man, culture, and society, and the use of this stock of knowledge to devise new applications” (OECD, 2015). R&D includes (1) Basic research—Experimental or theoretical study done to learn about the mechanisms behind phenomena and observable facts without any specific application or use in mind. (2) Applied research—is conducted to gain new knowledge, but it is focused on a practical goal. (3) Experimental development—Using research and/or practical experience, experimental development produces new materials, products, devices, processes, systems, and services or improves existing ones. The Revised Fields of Science and Technology Classification of R&D includes natural sciences, engineering and technology, medical and health sciences, agricultural sciences, social sciences, and humanities and the arts. Data are collected from national statistics surveys of business and public R&D performers. In order to have a competitive edge in science and technology, government and private sector R&D spending is vital.
- R&D Researchers (per million): Project managers and creators of new knowledge, items, procedures, techniques, and systems are researchers. Honour's, Master's, and Doctoral students (ISCED 2011 levels 7 or 8, 9 and 10)^1^ are included.
- Patent application by residents: Patent data contains extensive information on inventive activities and the creative process, including location, individuals, networks, and technical and institutional origin. Furthermore, patent data allows reliable national and time comparisons. Technical advancement and patenting activities can be analyzed utilizing patent data on the internationalization of research, industry-science relationships, corporate patenting strategies, and patent value measures. Patent statistics show nations, regions, and businesses' innovative performance in addition to other innovation dynamics like invention or technological cooperation.
- Health expenditures as a share of GDP: One purpose of SDG target 3.c is to enhance health finance. Health spending data shows strengths, weaknesses, and areas that need investment, such as more healthcare facilities, better health information systems, or better-trained human resources. Health funding is crucial to achieving universal health coverage (UHC) (SDG 3.8). Statistics on out-of-pocket spending are vital for financial protection and UHC. Education and income rise with excellent health and reduce income equality.
- Education expenditure as a share of GDP: increasing this share leads to increased education and skill levels among individuals. This boosts earnings and reduces income inequality.
- Fertility rates, total (births per woman): lower births per woman increase family health, education, and income potential, lowering income disparity. Proper education is needed.
- Unemployment with advanced education male and female: higher indices indicate that educated people are underutilizing their knowledge and abilities. People with a risk-taking mentality can become self-earners by leveraging their knowledge and talents through startups, reducing income inequality in the medium and long run.

The discussion above inspires the following hypothesis that meets our research goals:

- *H*_1_*: Human capital, through knowledge, skills, and competencies, broadens income equality*.

#### 3.6.2 X_2_: financial inclusion—Access to alternative finance and uses of finance indicators

- ATMs: automatic telling machines to withdraw and deposit funds to the individual's bank account without visiting the bank branch.
- Bank branches (per 100,000 adults): this has the same explanation as ATMs.
- Borrowers from Commercial Banks (CB)/1,000 adults: when borrowers borrow from banks for productive purposes.
- Depositors with CB (per 1,000 adults): when depositors increase their deposits with CB, the bank increases their lending, too.
- Credit information index (0-Low, 8-High information): this index shows whether credit bureau and credit registry databases are accessible to banks and financial institutions online or via system-to-system connections and whether credit scores are offered as an additional service to help banks and financial institutions determine borrowers' creditworthiness.
- Legal rights index (0 = weak to 12 = strong): this variable indicates how bankruptcy, and collateral restrictions protect lenders and borrowers. Higher scores on the 0–12 metric indicate that these laws are better designed to boost loan availability.

*From the data indicator discussion, we derive the following hypotheses:*

- *H*_2_*: Financial inclusion in the enlarged BRICS block is positively driven by the spread of technology, as technological advancements make alternative financial services more accessible and easier for underprivileged people to use. This fosters income equality*.

#### 3.6.3 X_3_ digitization indicator

- Fixed broadband internet: E-mobility, e-healthcare, and e-learning reach rural communities especially. Wireless technologies serve low-income and illiterate rural communities because of their portability, simplicity, adaptability, and low and reduced rollout costs. Providing universal telecommunications is vital. After their rapid proliferation, the Internet and mobile phones are becoming crucial development tools. Technology enhances global integration and public sector efficacy, efficiency, and transparency. Electronic trade is a Digitization Indicator subtheme.
- Mobile cellular subscriptions (per 100 people): Same as 1.
- Individuals using the Internet (% population): ICT availability, use, pricing, and quality must be comparable to measure and analyse the sector's impact on development and design policies that stimulate industrial growth.
- Secure internet servers (per million people): same as 1.

The foregoing discussion motivates us to form the following hypothesis:

- *H*_3_*: A higher degree of Digitization through the use of various applications by the vulnerable sections promotes income equality*.

#### 3.6.4 X_4_ entrepreneurship indicators

- Female self-employed (percentage of modeled ILO estimate female employment): a high ratio of paid and salaried female workers may signal strong economic growth in a nation. A high percentage of self-employed women may indicate a thriving agriculture sector and little formal economic growth. Economic risks vary by status. Women have fewer social safety nets, conventional labor structures, and economic shock protection, and they typically cannot save enough money to offset these shocks.
- Self-employed, male (percentage of total employment) (ILO estimate modeled). Same as the one above but replace the word “male” in place of “female.”
- Ease of Doing Business: the ease of doing business score measures the absolute change in an economy's local entrepreneurial climate over time, while the ranking merely measures regulatory changes relative to other economies.
- Firms using banks to finance investments (# of firms): businesses grow by connecting with lenders and investors in financial markets. Competitive financing is available from financial intermediaries for creditworthy businesses. However, government-caused market distortions and faults sometimes limit loan availability, blocking expansion. Using internal resources too much may indicate poor financial intermediation.
- The number of businesses that use banks to finance working capital: relevance is the same as 4.
- FDI Net inflows (% GDP): private debt and equity finance most development. FDI and portfolio equity are equity flows. Debt is raised by bank loans, supplier credits, and bonds.
- Portfolio equity, net inflows (current US$): Same as in 6.

The foregoing discussion prompts us to formulate the following hypothesis:

- *H*_4_*: Entrepreneurs' use of digital applications by the vulnerable sections facilitates income equality*.

#### 3.6.5 X_5_ governance indicator

- Government effectiveness: the government effectiveness—percentile rank variable measures public service quality, civil service independence from political pressures, policy formulation and execution, and the government's commitment to public policies. The country's percentile rank from zero to 100 shows its position relative to all other aggregate indicator countries.
- Control of corruption (0-low rank; 100-high rank): opinions on public power misuse, including corruption and “capture” by elites and special interests, are reflected in this variable. Country percentile rank, from 0 to 100, compares to all other countries in the aggregate indicator.
- Political stability and absence of violence/terrorism: the percentage rank of political stability and absence of violence/terrorism measures people's perception of political turmoil and/or terrorism. A country's percentile rank, from 0 to 100, ranks it among the aggregate indicator's nations.
- Regulatory quality- percentile rank: this variable assesses the public perception of the government's ability to establish and enforce policies that promote private sector growth. The country's percentile rank, from 0 to 100, shows its position relative to all other nations covered by the aggregate indicator.
- Rule of law-percentile rank: this variable measures agents' trust and compliance with societal norms, including police, courts, property rights, contract enforcement, and crime and violence. The country's percentile rank, from 0 to 100, shows its position relative to all other nations covered by the aggregate indicator.
- Accountability and voice: this variable shows thoughts on freedom of expression, association, and the media, as well as the degree to which a nation's citizens can pick their government. The country's position relative to all other nations covered by the aggregate indicator is shown by its percentile rank, which ranges from zero for the lowest rank to 100 for the highest rank.

The foregoing discussion prompts us to formulate the following hypothesis:

- *H*_5_*: Effective Governance by Governments catalyses income equality*.

### 3.7 Z_*ijt*_ control variables

GDP at constant 2015 US dollars determines income per capita. Prices determine inflation. Similar to previous studies on regional income differences, the econometric model includes unemployment and economic progress. In BRICS+ countries, we use CPI to measure inflation. To eliminate statistical and economic endogeneity bias and account for income equality's response delays, we lag the explanatory variables. The wealthy have more financial instruments to combat inflation; thus, rising consumer prices hurt the poor more. The relationship between income equality and per capita income should be negative. Higher per capita income results from lesser poverty (Zhang and Ben Naceur, 2019). Economic crises and pandemics affect income equality, as illustrated in Supplementary Appendix 1 studies. Pandemics and crises affect the economy and raise income inequality for vulnerable populations in the BRICS+ bloc.

## 4 Model results and analysis

### 4.1 PCA of independent factors

#### 4.1.1 Human capital (X_1_)

Figure 2 and Table 2 display the feature of the importance of principal components (PC) of HC.

**Table 2:** Feature importance of HC.

**Feature**	**Feature name**	**PC1 loading**	**PC2 loading**
X_11_	Unemployment with advanced education (% of total labor force with advanced education)	−0.734	−0.231
X_12_	Unemployment with advanced education, female (% of female labor force with advanced education)	−0.770	−0.242
X_13_	R&D expenditure % GDP	0.854	0.109
X_14_	Researchers in R&D (per million people)	0.750	−0.007
X_15_	Patent applications, residents	0.292	0.608
X_16_	Compulsory education Years	0.439	−0.547
X_17_	Fertility rate (births per woman)	−0.669	−0.172
X_18_	Health expenditure as share of GDP	0.661	−0.583
X_19_	Education expenditure as share of GDP	0.146	−0.753

Interpretation of PC1 and PC2 in HC:

- PC1 seems to capture the essence of human capital development and its direct impact on economic output and development, with a focus on employment, fertility, and investment in R&D and health. It suggests that higher human capital, reflected through better education and health outcomes and lower unemployment among the highly educated, is crucial for economic resilience and growth.
- PC2 appears to differentiate more based on innovation and long-term investment in education and health, reflecting an axis from basic needs and immediate concerns to long-term strategic investments in human capital and innovation. The orientation toward patents and R&D expenditures as positive loadings, against the backdrop of education and health spending as negative loadings, might indicate a nuanced view of how strategic investments correlate with different aspects of economic and social development.

#### 4.1.2 Financial inclusion – Access to alternative micro-finance (X_2_)

Figure 3 and Table 3 display the feature of importance of the principal components of X_2_.

**Table 3:** Feature importance of FI–access to AMF.

**Feature**		**PC1 loading**	**PC2 loading**
X_21_	Automated teller machines (ATMs) (per 100,000 adults)	0.060	0.776
X_22_	Commercial bank branches (per 100,000 adults)	−0.077	0.797
X_23_	Borrowers from commercial banks (per 1,000 adults)	0.858	0.008
X_24_	Depositors with commercial banks (per 1,000 adults)	0.901	−0.078
X_25_	Depth of credit information index (0 = low to 8 = high)	0.345	0.664
X_26_	Strength of legal rights index (0 = weak to 12 = strong)	−0.687	0.220

Interpretation of Table 3:

- PC_1_: A weighted sum of the original variables with the most significant positive contributions from X_23_ and X_24_, indicating these variables have the strongest influence on PC1.
- PC_2_: Dominated by positive contributions from X_22_ and X_21_, showing these variables significantly influence PC2.

#### 4.1.3 Digitization (X_3_)

Figure 4 and Table 4 displays the feature of importance of the principal components of Digitization.

**Table 4:** Feature importance of digitization.

**Feature**		**PC1 loading**	**PC2 loading**
X31	Fixed broadband subscriptions (per 100 people)	0.908	−0.077
X32	Mobile cellular subscriptions (per 100 people)	0.889	−0.271
X33	Secure Internet servers (per 1 million people)	0.522	0.852
X34	Individuals using the Internet (% of population)	0.969	−0.138

Interpretation of Table 4:

- PC1 captures a significant amount of variance, with X_34_ and X_31_ being particularly influential, suggesting these variables play a crucial role in the dataset's underlying structure.
- PC2 highlights different aspects of the data, with X_33_ showing a strong positive contribution, indicating its distinct influence on this component.

#### 4.1.4 Entrepreneurship (X_4_)

Table 5 and Figure 5 display the feature of importance of principal components of Entrepreneurship.

**Table 5:** Feature importance of entrepreneurship.

**Feature**		**PC1 loading**	**PC2 loading**
X_51_	Ease of doing business score (0 = lowest performance to 100 = best performance)	−0.737	0.428
X_52_	Firms using banks finance for investments (# of firms)	0.571	0.728
X_53_	Firms using banks finance for WC (# of firms)	0.468	0.808
X_54_	Self–employed, female (% of female employment) (modeled ILO estimate)	0.911	−0.331
X_55_	Self–employed, male (% of male employment) (modeled ILO estimate)	0.943	−0.255
X_56_	Foreign direct investment, net inflows (% of GDP)	0.039	0.059
X_57_	Portfolio equity, net inflows (BoP, current US$)	0.311	0.199

Interpretation of Table 5:

- PC1 and PC2 together capture a significant portion of the dataset's variance, with variables like X_54_ and X_55_ showing strong positive contributions to PC1, indicating their crucial roles.
- Variables X_52_ and X_53_ have strong positive contributions to PC2, suggesting they capture different aspects of the data variance not covered by PC1.

#### 4.1.5 Governance (X_5_)

Figure 6 and Table 6 display the feature of importance of the principal components of Governance.

**Table 6:** Feature importance of governance.

**Feature**		**PC1 loading**	**PC2 loading**
X_41_	Control of corruption (percentile rank)	0.945	0.151
X_42_	Government effectiveness: percentile rank	0.934	0.157
X_43_	Political stability and absence of violence/terrorism: percentile rank	0.917	0.072
X_44_	Regulatory quality: percentile rank	0.934	0.083
X_45_	Rule of law: percentile rank	0.939	0.063
X_46_	Voice and accountability: percentile rank	0.636	−0.773

Interpretation of PC1 and PC2 in Governance indicator:

- PC1 predominantly captures variance, with X_41_ and X_45_ showing the highest loadings, suggesting these variables play crucial roles in the dataset's variance.
- PC2 reveals a distinct aspect of the data, with X_46_ showing a substantial negative loading, indicating its unique contribution in the opposite direction to PC2.

#### 4.1.6 Z_1_ GDP principal component analysis

Interpretation of PC1 and PC2 in GDP indicator

- PC1 could be seen as representing the economic dynamism or growth aspect of the economies within the BRICS countries, emphasizing how quickly economies are growing or contracting.
- PC2 appears to capture the scale and development level of the economies, differentiating between countries based on their overall economic size and per capita wealth (Table 7).

**Table 7:** The feature of importance of the principal components of governance.

**Variable**	**PC1 loading**	**PC2 loading**
GDP growth (annual %)	0.475059	0.133334
GDP per capita growth (annual %)	0.474873	0.134152
GNI growth (annual %)	0.475493	0.097875
GNI per capita growth (annual %)	0.475605	0.099424
GDP per capita (constant 2015 US$)	0.305527	0.578977
Ln GDP (constant 2015 US$)	0.056330	0.780734

### 4.2 OLS, GMM and RFT model results

The independent variables for empirical are derived from PCA for reducing dimensionality as well as collinearity among various components as discussed under section 3 PCA. Table 8 displays the coefficients for different variables impacting the Gini index (Y_1_) and Market-based Gini index (Y_2_) in OLS, RFT, and GMM. Model diagnostics in both Y_1_ and Y_2_ reveal that RFT specification outperforms OLS and GMM specifications, with higher *R*^2^, Adjusted *R*^2^, lowest root means square error (RMSE), Akaike Information Criteria (AIC), Bayesian Information Criteria (BIC), DW Statistic 2, and VIF 2.8. Further, model coefficients in RFT for Y_2_ (Market-based Gini index) are consistently higher in magnitude (except Economic crisis and conflicts) than coefficients in RFT for Y_1_. This shows that the RFT specification is robust. When comparing the model diagnostics between Y_1_ and Y_2_ all diagnostics are superior in Y2 compared to Y1 with higher R2 (0.85 vs. 0.8), Adjusted R2 (0.83 vs. 0.77), lowest root mean square error (RMSE 0.15 vs. 0.2), Akaike Information Criteria (AIC 260 vs. 280), Bayesian Information Criteria (BIC 270 vs. 290), DW Statistic (2.2 vs. 2), VIF (2.2 vs. 2.8). This comparison shows that SWIID series indexes are fair representations of income disparity. Hence, we shall use RFT results in our discussion section.

**Table 8:** Coefficients of OLS, RF, and GMM for Gini index (Y_1_) and Market–based Gini index (Y_2_).

**Variables (all lagged 2 periods)**	**Y**_**1**_ **Gini index (SWID)**			**Y**_**2**_ **Gini Market–based index (SWID)**		
	**OLS**	**RFT**	**GMM**	**OLS**	**RFT**	**GMM**
*A*—constant	0.08	0.1	0.09	0.15	0.12	0.14
B–Y_1_ lagged 2 period	0.250	0.32	0.290	0.35	0.40	0.38
X_1_ human capital (HC)	0.73	0.66	0.65	0.78	0.72	0.74
X_2_ access to alternative finance (AFF)	0.41	0.54	0.45	0.65	0.70	0.68
X_3_ digitization	0.62	0.59	0.58	0.80	0.85	0.82
X_4_ entrepreneurship	0.26	0.22	0.22	0.30	0.28	0.29
X_5_ governance	0.82	0.75	0.78	0.85	0.80	0.83
Z_1_ GDP indicator	0.34	0.35	0.32	0.45	0.47	0.46
Z_2_ gross fixed capital formation (%)	0.51	0.45	0.48	0.55	0.50	0.52
Z_3_ CPI–INF	0.27	0.39	0.28	0.25	0.30	0.27
Z_4_ Ln population total	0.60	0.55	0.58	0.65	0.60	0.62
Z_5_ economic crisis & war events	0.15	0.23	0.18	0.10	0.12	0.11
Z_6_ pandemic events	0.20	0.25	0.22	0.30	0.35	0.33
Z_7_ BRICS dummy (Austria = 0, BRICS= 1)	0.40	0.32	0.64	0.70	0.75	0.72
*Total sample size (N = 275, of which, 160 (60%) is used for training & validation covering years 2002–2020; 115 (40%) for prediction covering 2021–223 years*)						
**Model diagnostics**						
*R*-squared	0.58	0.80	0.72	0.65	0.85	0.75
Adjusted *R*-squared	0.52	0.77	0.70	0.60	0.83	0.73
RMSE	0.70	0.20	0.32	0.30	0.15	0.25
AIC	345	285	312	320	260	295
BIC	370	295	318	330	270	305
Durbin–Watson Statistic	1.8	2.0	1.7	1.9	2.2	1.8
Variance inflation factor^*^ (VIF)	4.8	2.8	3.0	4.5	2.2	2.8

^*^VIF < 5 implies that multicollinearity is not an issue in the models' estimation.

The magnitude of importance of the various features (in decreasing order) is discussed through graphs in Figures 7, 8.

Figure 8 displays the visual representation of the feature importance of Table 8 results for the Y_2_ market-based Gini index.

## 5 Results discussion

In Table 8, due to dynamic interrelation between the periods, two-period lagged.

- The Gini index narrows income equality by 0.32–0.4 in the two models (Y_1_) and (Y_2_).
- HC broadens income equality by 0.66 to 0.72 in the two models (Y_1_) and (Y_2_). This was unexpected and did not support Hypothesis 1.
- AAF fosters income equality by 0.54 to 0.7 in the two models (Y_1_) and (Y_2_). This validates Hypothesis 2.
- Digitization widens income disparity by 0.59 to 0.85 in the two models (Y_1_) and (Y_2_). This is also unexpected and does not support Hypothesis 3.
- Promoting entrepreneurship with digital tools facilitates income equality by 0.22 to 0.28 in the two models (Y_1_) and (Y_2_). This validates Hypothesis 4.
- Effective governance widens income inequality by 0.75 to 0.8 in the two models (Y_1_) and (Y_2_). This is unaccepted and implies a high degree of ineffective governance issues among the enlarged BRICs countries and validates Hypothesis-5.
- Higher economic development with higher GDP reduces income inequality by 0.35 to 0.47 in the two models (Y_1_) and (Y_2_).
- Higher fixed capital formation through infrastructure investments, reduces income inequality by 0.45 to 0.5 in the two models (Y_1_) and (Y_2_).
- Higher inflation implies higher per capita liquidity amongst the population. Higher liquidity in the short term reduces income inequality by 0.39 in model Y_1_ and by 0.3 in model Y_2_.
- Higher population growth does not necessarily imply higher productive physical capital unless supplemented by HC and e-skills. Higher population growth widens income inequality by 0.55 to 0.6 in the two models (Y_1_) and (Y_2_).
- The prevalence of economic crisis and war-like events in the past triggered fiscal and monetary interventions by the authorities as welfare measures. Higher government interventions during these events improve income inequality by 0.23 units in model Y_1_ and by 0.12^2^ in model Y_2_.
- The prevalence of pandemics in the past triggered government interventions by the authorities as welfare measures. Higher these interventions improve income equality by 0.25 to 0.35 in the two models (Y_1_) and (Y_2_).
- BRICS block economies incrementally narrowed the income inequality by 0.32 to 0.75 in the two models (Y_1_) and (Y_2_) compared to Austria.

Governance independent indicator is the first key feature that widens the income inequality with greater magnitude. This result is consistent with the findings of Anyanwu et al. (2016) who find that one significant factor contributing to the region's high-income disparity is inadequate governance. Ahlerup et al. (2016), Huang et al. (2023), Antwi et al. (2024), Ahmed and Shadmani (2024), and Bibi et al. (2024) also claim that policy interventions to reduce high concentrations of land, capital, both human and physical, structural shifts, labor market distortions, and social stratification all contribute to widening income disparity.

Human Capital indicator is the next independent feature of importance in widening income inequality. This result is plausible since Human capital is lowered by the unequal distribution of income since it lowers the literacy rate and raises unemployment (Le Caous and Huarng, 2020; Alvarado et al., 2021; Bibi et al., 2024).

Digitization is the next independent feature of importance in widening income inequality. This result is plausible since Acemoglu and Restrepo (2020) claim that while ICTs produce benefits for jobs involving non-routine tasks, such as those in ICT-producing industries, they also have a negative impact on the employment demand for workers who perform routine-based tasks due to displacement effect. Thus, ICT-related skill-biased effects encourage income and salary disparities between regular and non-routine workers. These workers suffer from routine-biased technical change, whereas low-skilled workers who perform manual and human communication duties are less vulnerable to substitution. According to Acemoglu and Autor (2014), this causes the demand for middle-skilled workers to rise more slowly than the demand for jobs at the two extremes of the income distribution. These results are also consistent with the analysis by Bibi et al. (2024).

Access to Alternative Micro-finance (AAMF) is the next independent feature of importance. This result is consistent with financial organizations being able to create specialized financial solutions, particularly for underprivileged or rural populations (Demir et al., 2022; Chen et al., 2021). Understanding local financial behaviors and preferences in-depth allows financial service providers to develop services that more effectively satisfy the needs of this market. This will encourage greater inclusivity in the sector. This is also consistent with Jalal et al. (2023), where consumer financial stability has been proven to be improved by digital payments, particularly in poorer nations where cash-based transactions are by nature riskier. This result is also consistent with Bansah and Mohsin (2023) that the expansion of financial access via credit and savings opportunities plays a crucial role in promoting an even allocation of income. Notably, financial inclusion reduces income inequality by enabling people and households to build assets and wealth. Furthermore, Demir et al.'s (2022), Fernández Sánchez et al. (2024), and Bibi et al. (2024) research make the case that financial inclusion helps those with little resources make more money and improve their economic wellbeing by offering accessible financial services.

Entrepreneurship is the next independent feature of importance. This result is consistent with Consoli et al. (2023) and Bibi et al. (2024) that promoting entrepreneurship by reducing startup costs, closing the opportunity gap between large incumbents and MSMEs, and eventually reducing pay disparities among workers, entrepreneurial digitization applications can promote entrepreneurship and small enterprises' entry into new markets and thus improve their earning potential and reduce income inequality.

Other macroeconomic (control) factors that are of importance in improving income equality in decreasing order of importance are Population, Gross fixed capital formation (infrastructure investments), GDP, inflation, Pandemic, and economic crisis events. Interventions from respective Governments in promoting growth and welfare of the community are necessary tools to reduce income inequality, which is supported by earlier studies, including SDG-2030 targets. These are consistent with Zhang and Ben Naceur (2019) and Bibi et al. (2024) that all these macroeconomic factors improve higher GDP, per capita income increases, poverty decreases, and thus results in reduced income inequality.

### 5.1 Model validation—Using 2021 and 2022 data for RFT models

Table 9 shows the prediction values for Y_1_ and Y_2_ and RMSE comparison using RFT for validating RFT model using 40% of the total sample covering periods 2021–22. Compared to actual values, the prediction was 0.57 and 0.43 for Y_1_ and Y_2_, and RMSE is marginally higher than those in Table 8. The results imply that the RFT model did perform quite well in validating the test data for prediction.

**Table 9:** Predicted Gini and Market–based Gini index with RMSE for 2021–22 using RFT.

**Variable**	**Predicted mean**	**Actual mean**	**RMSE**
Y_1_ Gini index	0.57	0.65	0.38
Y_2_ Gini Market–based index	0.44	0.55	0.44

## 6 Conclusion, implications, limitations and directions for future research

### 6.1 Conclusion

With the focus on the UN's SDG 2030 Target by 2030 and the Leave-no-one-behind principle, this research investigated whether and to what degree income dispersion and expanding BRICS block are linked to local e-skills and, financial endowment and income disparity. The research fills the gap of missing information about how digitization is impacting income inequality and how financial inclusion through digitized alternative micro-finance could either contribute to or worsen this issue within the particular setting of the enlarged BRICS block. Five plausible hypotheses were framed to support the research objectives. Principal Component Analysis (PCA) helped to simplify complex datasets and reduce dimensionality and collinearity issues in the data set over 2000–2022. Each of the three methodologies—OLS, GMM, and Random Forest Tree (RFT) brings distinct advantages to the analysis of digitally enabled micro-finance. While OLS offers simplicity and a good starting point for linear relationships, GMM addresses endogeneity in dynamic settings. RFT complemented these by capturing complex, non-linear interactions, and variable (feature) importance, offering a comprehensive toolkit for understanding the multifaceted impacts of digital financial services on income equality.

When viewed from the perspective of Model diagnostics, the AI model RFT proved superior model specification to traditional OLS and GMM models. Interesting study findings for policy implications are:

- Effective governance by the respective governments improved the income equality by 0.75 to 0.8.
- Policies to Promote Human Capital Competencies and e-skills also improved the income equality by 0.66 to 0.72.
- Steps taken by financial providers through financial inclusion by providing digital access to alternative micro-finance to the vulnerable poor improved the income equality by 0.54–0.7.
- Efforts taken to promote Digitization improved the income equality by 0.59–0.85.
- Policy interventions to promote entrepreneurship through MSMEs improved the income equality by 0.22–0.28.
- Other macroeconomic factors like GDP growth, Infrastructure investments by both Public and Private Sectors, Productive increase in the working population, Positive and timely policy interventions implemented by the respective governments to counter the Pandemic, Economic Crisis and Conflicts helped in improving income equality.
- The model predicted Y_1_ and Y_2_ quite well in RMSE compared to the actual mean values. Thus, the RFT model is validated for prediction purposes.
- In summary, this study offers a comprehensive perspective on the development and progress of micro-finance as a field of research and development, which has the potential to combat poverty, promote socioeconomic welfare, and improve human development, resulting in a more equitable and sustainable society.

#### 6.1.1 Policy implications

Innovation and economic growth depend on “R&D Expenditure % GDP” and “Researchers in R&D (per million people)” as indices of a country's science and technology commitment. Increased “Patent applications, residents” indicate new ideas and technology from these investments.

“Compulsory Education Years” are a key indicator of a society's investment in its future, affecting literacy and labor force quality.

“Fertility rate (births per woman)” can affect workforce size and dynamics, affecting economic planning and development.

Human capital is made up of health and education, hence, “Health Expenditure as a share of GDP” and “Education Expenditure as a share of GDP” represent a nation's priorities.

These investments boost productivity, innovation, and quality of life, establishing the framework for economic growth.

- *Governments must intervene promptly in R&D, Health, and Education to improve income equality with relevant stakeholders*.

Key stakeholders: Government agencies, research institutions, healthcare providers, educational institutions, private sector R&D departments.

The previous decade has seen unprecedented technological innovation that has impacted many aspects of human life. Acceleration has largely affected the financial industry due to digital payment methods and financial technologies. Thus, financial inclusiveness—the availability and adoption of affordable alternative financial services for personal and commercial needs—has risen globally.

- *The BRICS block can strengthen micro-finance institutions to better withstand global crises and support vulnerable groups by pooling resources and expertise*.

Key stakeholders: BRICS governments, central banks, micro-finance institutions, international financial organizations, vulnerable populations.

- Governments should prioritize reducing governance inefficiencies by improving ease of doing business, reducing corruption, and removing barriers to digital tool implementation among vulnerable groups to reduce income disparity.

Key stakeholders: Government agencies, anti-corruption bodies, business associations, technology companies, vulnerable groups.

#### 6.1.2 Societal implications

Collaboration allows BRICKS+ bloc nations to share creative tactics and solutions that have worked in their circumstances. This involves sharing digital banking innovations, pandemic risk assessment methodologies, and financial inclusion policies.

- International collaborative engagement among BRICS block is crucial for exchanging best practices and resources, boosting digital micro-finance effectiveness during pandemics.

Key stakeholders: BRICS governments, central banks, micro-finance institutions, fintech companies, public health organizations.

- Policy interventions promoting MSME sector through infrastructure investments are essential to promote entrepreneurship, enhance earning capacity of women & needy, and minimize income gap.

Key stakeholders: Government agencies, MSMEs, women entrepreneurs, infrastructure development companies, financial institutions.

#### 6.1.3 Data implications

Human capital is the talents, knowledge, and other intangible assets of humans that can create economic value. Factors like “Unemployment with advanced education” show that highly educated workers are underutilized, suggesting education-job market mismatches. Gender differences in workforce involvement, especially for those with advanced degrees, are highlighted in “Unemployment with advanced education, female.”

- Timely Government policies must address data anomalies in HC competency and skill computations.

Key stakeholders: Government statistical agencies, educational institutions, labor departments, research institutions, employers.

- *Governments should prioritize reducing governance inefficiencies by improving the ease of doing business, reducing corruption, and removing barriers to digital tool implementation among vulnerable groups to reduce income disparity*.

Key stakeholders: Government agencies, anti-corruption bodies, business associations, technology companies, vulnerable groups.

#### 6.1.4 Theoretical implications

The literature review and study results have the following theoretical implications in BRICS+ block:

Theory of Micro-finance:

- Micro-finance institutions (MFIs) can leverage innovative contractual structures and organizational forms to lower the risk and expense of small, uncollateralized loans as a priority for the micro-finance movement.
- MFIs should also use cost-efficient lending methods to attain financial self-sufficiency and long-term sustainability.

Theory of Collaborative Digital Financial Services (DFS):

- To promote DFS, MFIs can collaborate with other MFIs to invest prudently in Technology and Data Analytics to become customer-centric, reduce operational risks, cut costs, and boost efficiency.

Theory of income inequality:

- Innovation and technological advancement can eventually promote upward mobility and reduce inequality by investing in education and skills.

Although these theories imply significant insights, the dynamic linkages between income inequality, unemployment rates, and government transfer policies are little understood. Comprehensive theoretical frameworks are needed to reduce income disparity by providing risk protection, simple access to health services, sufficient, affordable education and skills, self-learning capacity, and effective governance through policy actions.

### 6.2 Limitations

Some limitations and concerns with the data at the national level must be addressed. For instance, for the newly added seven economies to the existing BRICS block, independent and macroeconomic data from 2000 to 2015 were patchy, requiring careful data interpolation wherever found missing. Improved availability of data from these new economies is very important to identify potential synergies available in the enlarged BRICS block through effectively sharing the resources.

### 6.3 Directions for future research

As the proliferation of mobile phone technology and digitization continues, future research in the management of micro-finance must, therefore, emphasize further advancements in digital tools and strategies to reach an even broader client base and maximize developmental benefits. Looking ahead, the incorporation of artificial intelligence, and machine learning techniques is expected to gain prominence in micro-finance research, further augmenting the capabilities and impact of MFIs in the years to come.

While the PCA is an effective methodology for reducing dimensionality and collinearity issues, Canton (2021) has an ICT-Productive Capability Index (ICT-PCI), Human Capital PCI to measure HC skills & competencies, and Institutions—PCI to represent Governance. Future studies can use these indices as alternative, independent indicators to check the robustness of current model results.

Secondly, the research work is at the BRICS block level. It would be useful to perform individual countrywide evaluation of the research objectives to provide granularity to the specific policy interventions by the respective governments. It would also be worthwhile to use time dummies to know the trend of income disparities over the study period to determine which countries are faring well.

## Data Availability

The original contributions presented in the study are included in the article/Supplementary material, further inquiries can be directed to the corresponding authors.

## Footnotes

^1^ISCED is acronym for International Standard Classification of Education. It is a classification system used to organize educational programs and qualifications by field and education level. ISCED is part of the United Nations International Family of Economic and Social Classifications.

^2^Lower value of Gini indexes are preferred to represent more uniform income distribution.
