# Supplementary material for: How does digitally enabled micro-finance promote income equality for the vulnerable in the expanded BRICS block during the pandemic?
Source: Front Big Data. 2024 Dec 2;7:1417752. doi: 10.3389/fdata.2024.1417752 (PMC11647032; doi:10.3389/fdata.2024.1417752)
Supplement: Supplementary file 1 [file Data_Sheet_1.docx]

**Appendix-1 Measurement and sources details of variables**

| **Variable** | **Data Sources** | **Previous research** |
| --- | --- | --- |
| Income equality (Dependent Variable)   - Gini Disposable index (Y_1_) - Gini Market-based index (Y_2_) | Standardized World Income Inequality  Database (SWIID)  World Bank, Poverty, and Inequality Platform. Data are based on primary household survey data obtained from government statistical agencies and World Bank  http://pip.worldbank.org | Iacono and Ranaldi 2022.  Tassaeva 2021.  Furceri and Ostry, 2019.  Royuela, Veneri, and Ramos (2019). |

1. **Human Capital Skills Relevance to Income Equality**

| **Variable** | **Data Sources** | **Previous research** |
| --- | --- | --- |
| 1. Compulsory education Years | https://apiportal.uis.unesco.org/bdds. | Khan et al., 2021.  Menyelim et al., 2021.  Le Caous & Huarng, 2020.  Alvarado, et al (2021);  Kunawotor, Bokpin, & Barnor, (2020).  Paul Cammack, 2017. |
| 1. Research and development expenditure as share of GDP | https://apiportal.uis.unesco.org/bdds. |  |
| 1. Researchers in research and development/ million people | https://apiportal.uis.unesco.org/bdds. |  |
| 1. Patent applications, residents | World Intellectual Property Organization (WIPO), WIPO Patent Report: Statistics on Worldwide Patent Activity. |  |
| 1. Health expenditures as share of GDP | WHO Global Health Expenditure database (http://apps.who.int/nha/database). |  |
| 1. Education Expenditure as share of GDP | World Development Indicators database |  |
| 1. Fertility rate | http://apps.who.int/nha/database |  |
| 1. Unemployment with advanced education male (%total labor force with advanced education | International Labor Organization. “ILO modelled estimates database” ILOSTAT. https://ilostat.ilo.org/data |  |
| 1. Unemployment with advanced education female (% total labor force with advanced education) |  |  |

II. **Financial Inclusion (FI) - Access to Alternative Micro-finance Relevance to Income Equality**

| **Variable** | **Data Sources** | **Previous research** |
| --- | --- | --- |
| 1. ATM /100,000 | IMF – FI-Access to Finance  IMF’s FAS website data.imf.org | Asif, et al. 2023; Konte, & Tetteh (2023); Shaikh et al. (2023); Jalal, Mubarak, & Durani 2023; Bansah, & Mohsin (2023); Bekele, 2023; Biyase, & Chisadza (2023); Coffie et al. 2023; Yin, & Choi, (2023); Park, & Mercado 2021; Huang et al. 2023; Demir et al., 2022; Chen et al. (2021). Goswami et al. 2022; World Bank (2022); Kim, (2022); Koomson, & Danquah (2021); Hasan, Yajuan, L., & Khan (2022); Kass-Hanna, Lyons, & Liu 2022; Kouladoum Wirajing, & Nchofoung 2022; Demirgüç-Kunt (2022); Lashitew, van Tulder, & Liasse (2019); Yu, & Tang 2023.  Wang, Li, & Li, 2023. |
| 1. Commercial bank branches per 100,000 adults | Same as above |  |
| 1. Borrowers from commercial banks (per 1,000 adults | IMF - Access to Finance  IMF’s FAS website data.imf.org |  |
| 1. Depositors with commercial banks (per 1,000 adults)( | Same as above |  |
| 1. Depth of credit information index (0=low to 8=high)( | Same as above |  |
| 1. Strength of legal rights index (0=weak to 12=strong) | World Bank, Doing Business project (http://www.doingbusiness.org/). |  |

1. **Digitalization (Infrastructure-ICT) relevance to Income Equality**

| **Variable** | **Data Sources** | **Previous research** |
| --- | --- | --- |
| 1. Fixed broadband subscriptions (per 100 people) | International Telecommunication Union (ITU) World Telecommunication/ICT Indicators Database | Acemoglu & Restrepo (2020).  Castellacci, Consoli, & Santoalha 2020.  Poliquin 2020. Rückert, Cathles, & Nayyar 2020.Tewathia, Kamath, & Ilavarasan 2020.  Bauer and Latzer 2016. Jorgenson and Vu 2016. Acemoglu and Autor 2014 |
| 1. Mobile cellular subscriptions (per 100 people) |  |  |
| 1. Individuals using the Internet (% of population |  |  |
| 1. Secure Internet servers (per 1 million people) |  |  |

1. **Entrepreneurship (Starting own business-MSMEs**) **Relevance** **to Income Equality**

| **Variable** | **Data Sources** | **Previous research** |
| --- | --- | --- |
| 1. Self-employed, female (% of female employment) | International Labor Organization. “ILO modelled estimates database” ILOSTAT. https://ilostat.ilo.org/data/. | Aghion et al. 2019  Ahlerup, Baskaran, & Bigsten (2016).  Anyanwu et al. (2016).  Tchamyou, Asongu, & Odhiambo (2019). |
| 1. Self-employed, male (% of male employment) |  |  |
| 1. Ease of doing Business Variables | World Bank: http://www.doingbusiness.org |  |
| 1. Firms using banks to finance investment (% of firms) | World Bank, Enterprise Surveys (http://www.enterprisesurveys.org/). |  |
| 1. Firms using banks to finance working capital (% of firms) |  |  |
| 1. Foreign direct investment, net inflows (% of GDP) | IMF, International Financial Statistics and Balance of Payments databases, World Bank, International Debt Statistics, and World Bank and OECD GDP estimates. |  |
| 1. Portfolio equity, net inflows (BoP, current US$) |  |  |

1. **Governance Indicators relevance to Income Equality**

| **Variable** | **Data Sources** | **Previous research** |
| --- | --- | --- |
| 1. Government Effectiveness: Percentile Rank | www.govindicators.org. | Tchamyou, Asongu, and Odhiambo (2019).  Ahlerup, Baskaran, & Bigsten (2016).  Anyanwu et al. (2016). |
| 1. Control of Corruption (0-Low rank; 100-High rank) |  |  |
| 1. Political Stability and Absence of Violence /Terrorism: Percentile Rank |  |  |
| 1. Regulatory Quality: Percentile Rank |  |  |
| 1. Rule of Law: Percentile Rank |  |  |
| 1. Voice and Accountability: Percentile Rank |  |  |

1. **Control variables.**

| **Variable** | **Data Sources** | **Previous research** |
| --- | --- | --- |
| 1. GDP growth (annual %) | World Development Indicators (WDI)  World Bank national accounts data, | Zhang & Naceur, 2019. |
| 1. GDP per capita growth (annual %) |  |  |
| 1. GNI growth (annual %) |  |  |
| 1. GNI per capita growth (annual %) |  |  |
| 1. GDP per capita (constant 2015 US$) |  |  |
| 1. GDP (constant 2015 US$) |  |  |
| 1. Population, total | WDI database  WHO database | Edmond Berisha, Ram Sewak Dubey Icon & Orkideh Gharehgozli (2023).  Dong-Hyeon Kim, and Shu-Chin Lin (2023). |
| 1. Gross fixed capital formation (% of GDP) |  |  |
| 1. CPI - Inflation |  |  |
| 1. Pandemic |  |  |
